# Pro-Inflammatory Biomarkers Combined with Body Composition Display a Strong Association with Knee Osteoarthritis in a Community-Based Study

**DOI:** 10.3390/biom13091315

**Published:** 2023-08-28

**Authors:** Nader Tarabeih, Alexander Kalinkovich, Adel Shalata, Orabi Higla, Gregory Livshits

**Affiliations:** 1 Department of Morphological Studies, Adelson School of Medicine, Ariel University, Ariel 40700, Israel; nadertar@gmail.com; 2 Department of Anatomy and Anthropology, Sackler Faculty of Medicine, Tel-Aviv University, Tel-Aviv 6905126, Israel; alexander.kalinkovich@gmail.com; 3 The Simon Winter Institute for Human Genetics, Bnai Zion Medical Center, The Ruth and Bruce Rappaport Faculty of Medicine, Technion, Haifa 32000, Israel; adel.shalata@gmail.com; 4 Orthopedics Clinic, Clalit, Migdal HaMeah, Tel-Aviv 6203854, Israel; orabi@windowslive.com

**Keywords:** osteoarthritis, biomarker, body composition, inflammation

## Abstract

Knee osteoarthritis (KOA) is one of the most common progressive, age-dependent chronic degenerative joint diseases. KOA often develops as a result of a gradual articular cartilage loss caused by its wear and tear. Numerous studies suggest that the degradation of the knee joint involves inflammatory components. This process is also associated with body composition, particularly being overweight and muscle mass loss. The present study aimed to search for novel circulating KOA inflammatory biomarkers, taking into account body composition characteristics. To this aim, we recruited 98 patients diagnosed and radiologically confirmed with KOA and 519 healthy controls from the Arab community in Israel. A panel of soluble molecules, related to inflammatory, metabolic, and musculoskeletal disorders, was measured by ELISA in plasma samples, while several body composition parameters were assessed with bioimpedance analysis. Statistical analysis, including multivariable logistic regression, revealed a number of the factors significantly associated with KOA, independently of age and sex. The most significant independent associations [OR (95% CI)] were fat body mass/body weight index—1.56 (1.20–2.02), systemic immune-inflammation index—4.03 (2.23–7.27), circulating vaspin levels—1.39 (1.15–1.68), follistatin/FSTL1 ratio—1.32 (1.02–1.70), and activin A/FSTL1 ratio—1.33 (1.01–1.75). Further clinical studies are warranted to confirm the relevance of these KOA-associated biological factors. Hereafter, they could serve as reliable biomarkers for KOA in the general human population.

## 1. Introduction

Osteoarthritis (OA) is one of the most common joint diseases [1]. Over 500 million individuals are currently estimated to be affected by OA worldwide [2]. OA mainly affects the hips and knees as predominant weight-bearing joints [3]; over 22% of adults older than 40 experience knee OA (KOA). OA is characterized by cartilage degradation, subchondral bone remodeling, and synovial membrane inflammation [4,5]. However, the etiology and pathogenesis of the disease are still not fully understood. Old age, female sex, and obesity are primary OA risk factors, but their prognostic contribution is weak and controversial [6,7]. Therefore, there is a medical need to identify novel OA biomarkers as reliable clinical indicators that can also act as surrogate endpoints in clinical trials for disease-modifying drugs. Several circulating molecules have revealed associations with OA [8,9,10,11] but do not meet diagnostic reliability criteria [12,13].

Low-grade, chronic inflammation with age (also termed inflammaging) is considered one of the major predisposing conditions in the pathogenesis of age-dependent, chronic degenerative conditions, including OA [14,15,16]. The detection of synovitis, along with immune cell infiltration and overexpression of inflammatory mediators in the synovium, is prominent in early OA stages, even preceding the development of radiographic damage [17,18]. Chondrocytes, synoviocytes, and infiltrating immune cells release various pro-inflammatory cytokines and chemokines. In particular, it has been shown that obesity affects the inflammatory phenotype of synovial fibroblasts (SFs) in hand joints, with the elevated secretion of pro-inflammatory monocyte chemoattractant proteins 1, 2, and 3 (MCP-1-3) and leukemia inhibitory factor. It has differential anatomic-site-specific effects on the transcriptomic landscape [19]. Some inflammatory factors, mainly tumor necrosis factor α (TNFα) and interleukins 1β (IL-1β), IL-6, IL-15, and IL-17, are implicated in OA pathogenesis [16,20,21,22]. Selective anti-inflammatory antagonists have been applied in clinical trials with, however, primarily disappointing outcomes in OA patients [23]. The search for additional pro-inflammatory molecules involved in OA development revealed that myokine follistatin-like protein 1 (FSTL1) promotes cell proliferation and the expression of several inflammatory cytokines in cultured FLSs obtained from the synovial tissues of KOA patients [24]. In support, serum FSTL1 levels are significantly higher in KOA patients than in respective control subjects [25]. Furthermore, several members of the transforming growth factor beta (TGF-β) superfamily, which regulates immune responses and inflammation [26], have been implicated in OA pathogenesis. For example, activin A concentrations are elevated in KOA cartilage compared to normal cartilage [27], and its serum concentrations positively correlate with the incidence and severity of KOA [28]. Follistatin, which has some structural homology with FSTL1 [29], is a well-known antagonist of TGF-β superfamily proteins such as myostatin and activin A [30]. Upregulated follistatin expression in the synovium tissue of KOA patients is accompanied by reduced activin A-induced proliferation of synovial [31,32]. These observations suggest a link between FSTL1, follistatin, and activin A in the inflammatory development of KOA.

Accumulating data indicate that body composition, specifically obesity, may increase the risk of OA development and manifestation [33,34]. This association has been assessed by body mass index (BMI) [35], waist–hip ratio (WHR) [36], high-fat mass (FM), and FM/skeletal muscle mass (SMM) ratio, mainly in women [37]. In obese patients, enlarged adipose tissue is widely known to support chronic inflammation [38,39]. The relationship between obesity and KOA is not only mediated through physical overloading but also through the production of adipokines possessing pro-inflammatory activity [40,41]. This presumption has been tested by analyzing the association between the circulating leptin and adiponectin levels and OA manifestation at different joints; the results were inconclusive [42]. Visceral adipose tissue-derived serpin (vaspin) is a relatively new adipokine that demonstrates an association with obesity and type 2 diabetes [43]. Interestingly, however, its plasma levels have been found to be elevated in KOA [44].

In addition to the above findings, which dictated the direction of the current research, we employed ratios between biomarkers as more effective predictors than individual biomarkers. One of the “classical” applicable ratios is the low-density lipoprotein cholesterol (LDL-C) or high-density lipoprotein cholesterol (HDL-C)/total cholesterol (TC) ratio in the prognosis of myocardial infarction [45]. The adiponectin/leptin ratio has been proposed with respect to KOA risk prediction [46]. The follistatin/myostatin ratio is implicated in monitoring exercise-inducing skeletal mass increase [47,48]. The ratio of adipokine adipsin to MCP-1 shows an association with cartilage volume loss in the lateral compartment over time. Finally, the C-reactive protein (CRP)/MCP-1 ratio is associated with KOA symptomatology in obese subjects [49].

Taken together, the central aim of this study was to evaluate the extent to which KOA manifestation is associated with the combined statistical effect of pro-inflammatory factors, their ratios, and body composition characteristics.

## 2. Materials and Methods

### 2.1. Study Design and Ethics

The data were collected from 617 individuals (mean age 53 ± 9.5 years, range 40–75 years) enrolled in outpatient clinics in Sakhnin (Israel) from 2015 to 2023. All participants were from the ethnically and culturally homogeneous population of Israeli Arabs [50]. They provided complete medical records or consented to provide access to their medical records. A total of 98 clinically diagnosed and radiologically confirmed KOA patients were selected for the present study. KOA diagnosis was determined according to the clinical and radiological criteria of the American College of Rheumatology [51]. The control group of 519 participants (251 males and 268 females) without clinical/radiological evidence of KOA was selected from the same population during screening. All the individuals in the study sample (both affected and control) were screened and examined at the same time in the outpatient clinic. The control individuals were defined as healthy if they did not suffer from a chronic illness, in particular musculoskeletal/joint diseases.

The inclusion criterion for the KOA group was diagnosed KOA confirmed by an orthopedist. The exclusion criteria for both groups were traumatic disorders, tumors, rheumatoid arthritis (RA), previous knee injuries or joint infections, systemic inflammatory or autoimmune disorders, severe heart problems, and age < 40 years.

Regardless of their KOA status, all participants in the study sample were assessed by certified and experienced nurses. Demographic data, anthropometrics, body composition measurements, and blood samples were collected from each individual. This research was approved by the IRB-Helsinki Committee (Number: 042/2013K, Date: 4 November 2013) of the Meir Medical Center, Kfar Saba, Israel, and the Ethics Committee of Tel Aviv University, Tel Aviv, Israel. Written informed consent was obtained from all participants prior to their inclusion.

### 2.2. Anthropometric and Body Composition Assessment

Demographic, anthropometric, and body composition data were collected and recently described in detail elsewhere [50]. Body mass index (BMI) was measured in kg/m^2^ and waist–hip ratio (WHR) in mm/mm. Body composition parameters were assessed by the bioimpedance (BIA) method [52]. BIA is considered a safe, reliable, simple, accurate, and inexpensive method to assess various body composition parameters [53]. BIA analysis included fat mass (FM) and skeletal muscle mass (SMM) in kilograms. Because body mass components are interrelated and depend on body weight (WT), they were presented as ratios to body weight, i.e., FM/WT and SMM/WT.

### 2.3. Analysis of Soluble Markers

Venous blood samples were obtained by venipuncture following overnight fasting. Within one hour after collection, they underwent centrifugation at 1800× *g* for 15 min at 4 °C. Plasma fractions were separated and stored in aliquots at −80 °C until usage. Quantities of soluble markers were detected by ELISA using the DuoSet kits (R&D systems, Minneapolis, MN, USA) according to the manufacturer’s protocols. The detection limits were as follows: 46.9 pg/mL for follistatin, 0.3 ng/mL for FSTL1, 49.6 pg/mL for vaspin, and 4.1 pg/mL for activin A. The intra- and inter-assay coefficients of variation were between 2.3% and 8.6%, respectively. Due to the significant deviation in the respective distributions from the normality assumptions, the original measurements of vaspin, follistatin, and activin A were subjected to log-normal transformation to approximate normality prior to analysis. Blood biomarkers included neutrophil, lymphocyte, platelet counts, and high-sensitivity CRP (hs-CRP) levels. The systemic immune-inflammation index (SII) was calculated (platelets × neutrophils/lymphocytes ratio) as previously described [54].

### 2.4. Statistical Analysis

Statistical data analysis was conducted using Statistica 64 (TIBCO Software, Version 13.5). The identification of the major covariates (potential predictors) for KOA, including age, body composition variables, and plasma levels of soluble markers, was carried out by independent sample *t*-test and parametric or non-parametric (Mann–Whitney), ANOVA comparing affected and non-affected groups of individuals. The basic significance threshold level was set at *p* < 0.05 for all the statistical tests. Adjustment for age and sex was included in all analyses. Finally, multiple logistic regression analysis with KOA as a dependent variable was carried out to test the relative and independent association of the selected biomarkers.

## 3. Results

The basic descriptive statistics of all the variables, separated by sex and disease status, are presented in Table S1. The sample included 281 males and 336 females in a similar age range (52.28 ± 0.57 years vs. 52.83 ± 0.48 years, *p* > 0.05). However, a significantly higher proportion of women than men was diagnosed with KOA: 20.24% vs. 10.70% (*p* = 0.001). As expected, body composition variables pertaining to adipose tissue mass were significantly different between the sexes, with BMI (31.51 ± 0.29 vs. 28.30 ± 0.24, *p* < 0.0001) and FM/WT (0.39 ± 0.003 vs. 0.26 ± 0.003, *p* < 0.0001) greater in females, whereas males had higher WHR (0.95 ± 0.003 vs. 0.91 ± 0.004, *p* < 0.0001) and SMM/WT (0.36 ± 0.002 vs. 0.25 ± 0.001, *p* < 0.0001). The levels of activin A were significantly higher in females compared to males (1512.01 ± 111.08 vs. 1026.03 ± 73.47, *p* = 0.0005). However, there were no statistically significant differences between males and females in the mean levels of vaspin, follistatin, hs-CRP, and SII. The age and sex effects of the study variables that displayed significant sex differences and/or were age-dependent were taken into account (adjustment) in the following analyses.

Table 1 shows that KOA patients were significantly older than control subjects (56.16 ± 1.08 years vs. 51.90 ± 0.38 years, *p* = 0.00002). Regarding body composition parameters, BMI and FM/WT were significantly higher, whereas SMM/WT was significantly lower in KOA patients compared to controls. These differences remained significant after adjustment for age and sex. There was no difference in WHR between the KOA and control groups. The plasma levels of vaspin, follistatin, and activin A, as well as hs-CRP and SII index, were significantly higher in KOA patients compared to controls. These differences remained significant after adjustment for age and sex. There were no differences in FSTL1 levels.

When blood biomarker ratios were compared between the KOA and control groups, highly significant differences (*p* < 0.001) were detected for activin A/FSTL1, and follistatin/FSTL1. These differences remained highly significant after adjustment for age and sex (*p* < 0.001).

**Figure 1:**
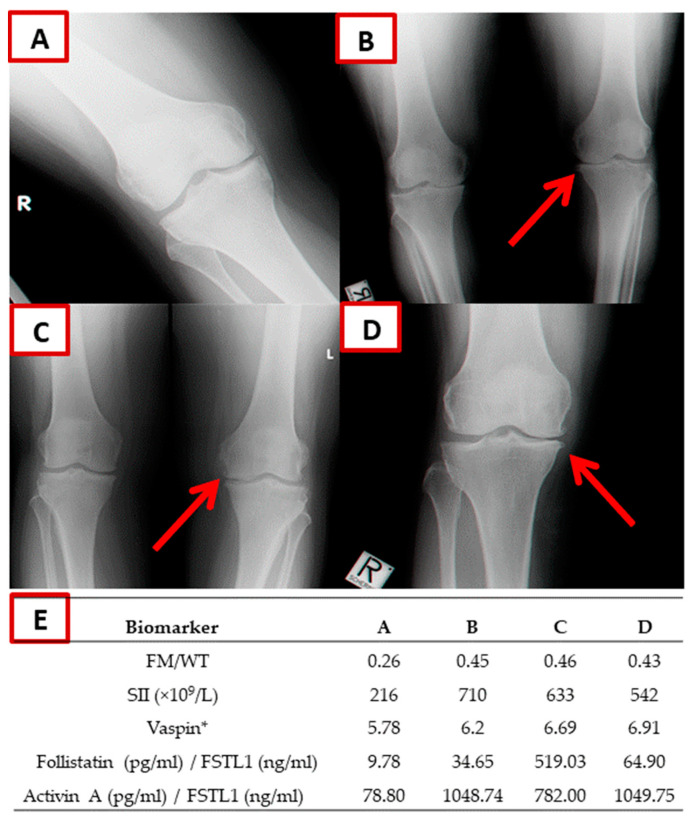
Examples of X-ray knee images. The figure shows two typical incidents of degenerative osteoarthritis of the knee joint in three individuals with KOA compared with a healthy individual. (**A**) A 55-year-old woman with a normal knee, and (**B**) a 53-year-old woman with severe KOA, accompanied by joint space narrowing in both legs at the medial aspect of the knee joint compared to its lateral aspect. Also, osteophytes are observed at the medial aspect of the knee joint (red arrow). In addition, sclerosis is observed in both knees. (**C**) A 51-year-old woman with severe KOA: joint space narrowing with osteophytosis in the left leg (red arrow); (**D**) a 55-year-old man with severe KOA in the left leg, accompanied by joint space narrowing and osteophytes (red arrow); and (**E**) the blood biomarker levels of these participants are presented in the table. * Plasma levels of the vaspin are presented after log-transformation to approximate normality prior to analysis.

Examples of KOA manifestations (radiograph scans) are given in Figure 1. The figure compares three KOA-affected individuals with one individual who has healthy joints. The table associated with the figure provides the values of tested biomarkers in each of the four individuals. KOA-affected individuals displayed higher levels of FM/WT, SII, vaspin, follistatin/FSTL1, and activin A/FSTL1 compared to the non-affected individual.

Next, we conducted multiple logistic regression analyses with KOA status as the dependent variable to investigate the combined and independent effects of covariates identified in the univariate context (Table 1). The results show that FM/WT, SII, plasma vaspin levels, and the ratios of follistatin/FSTL1 and activin A/FSTL1 were independently and significantly associated with KOA (Table 2). The calculated odds ratios (OR, 95% CI) ranged from 1.32 (1.02–1.70) for follistatin/FSTL1 levels to 4.03 (2.23–7.27) for SII. The likelihood ratio test for the model, including the effect of the significant covariate vs. a zero model, resulted in a highly significant estimate (*p* < 0.00001), thus confirming the reliability of the observed associations.

## 4. Discussion

OA, one of the leading causes of physical disability today, has become a major public health problem worldwide, with KOA being the predominant form of this disease [2]. Despite extensive research, the etiopathogenesis of OA remains unclear, and reliable prognostic biomarkers are not established. Growing evidence suggests that low-grade, chronic inflammation [15,55], often in combination with obesity [56], plays an important role in OA development. This motivated our search for new circulating KOA biomarkers by testing the association of KOA with several inflammatory markers in the blood and their ratios. Our main novel finding is the statistically significant and independent association of KOA with SII, follistatin/FSTL1, and activin A/FSTL1 ratios. We also confirmed the association of KOA with the plasma vaspin levels and FM/WT scores reported in other studies [37,44].

SII is a recently developed indicator applied to predict the risk of chronic inflammation disorders, such as atherosclerotic cardiovascular disease [57], osteoporosis [58], sarcopenia [59], inflammatory arthritides—psoriatic arthritis [60]—ankylosing spondylitis [60], and RA [61]. The association of SII with KOA, discovered in this study, is likely to be the first report on OA. Since synovial inflammation precedes structural joint degradation in OA [21], our data suggest that SII may become a simple and reliable prognostic biomarker for OA development. The validation of this finding is imperative.

Obesity is a well-established risk factor for KOA [33,34], and our data on a significant and independent association of FM/WT with KOA confirm this notion. In the adipose tissue of obese people, adipocytes undergo hypertrophy and hyperplasia, subsequently producing pro-inflammatory adipokines and cytokines, such as vaspin [38,62]. Vaspin is often linked to obesity and related metabolic dysfunction, e.g., glucose intolerance [63]. So far, vaspin has been only once tested for association with OA on a modest sample of 26 KOA patients and 23 healthy controls. The study found reduced serum vaspin levels in KOA patients [44]. The results of our study contradict this finding (Table 1 and Table 2); however, several other studies on inflammatory arthritides are aligned with ours. Higher plasma vaspin levels have been reported in patients with RA [64], psoriatic arthritis [65], facet joint OA and lumbar disc degeneration [66], and low back pain [50,67]. Still, from a metabolic viewpoint, it is unclear whether vaspin is a causative factor or if it plays a protective role in inflammation-associated disorders (reviewed in [68,69]).

In addition to adipokines, other soluble factors, in particular myokines, such as activin A [27,28,70] and FSTL1 [71], have been suggested to be involved in OA pathogenesis. The myokine glycoprotein follistatin, which shares some structural homology with FSTL1 [72] and acts as a decoy receptor for activins [73], has been recently suggested to be involved in OA pathogenesis as a protective factor [74,75]. In our study, the follistatin/FSTL1 ratio synergized their association with KOA diagnosis. It remained significant after adjustment for age and sex and other factors in a multivariable analysis. The pathophysiological nature of this finding is probably complex and might be related to the opposite activities of the follistatin and FSTL1. Follistatin possesses protecting activity on the development of arthritic manifestations [74,75]. In contrast, available data suggest detrimental activity of FSTL1 in joint diseases, including OA [24,76]. If such opposite functions of follistatin and FSTL1 are relevant for OA, an elevated follistatin/FSTL1 ratio can reflect disease progression rather than etiology.

Similarly, the activin A/FSTL1 ratio displayed a significant positive association with KOA and, therefore, also deserves elucidation of the possible underlying mechanism. Current data on the elevated expression of activin A suggest a potentially detrimental effect on joint health conditions [27,28,31,77]; although, contradictory results were also published [78]. The clinical significance of the activin A/FSTL1 ratio in KOA pathogenesis is unclear. However, possible metabolic relationships between activin A and FSTL1 have been reported. For example, FSTL1 is a distant homolog of follistatin [72], which acts as the antagonist to activin A [73,79]. In this regard, it is worth mentioning that follistatin, FSTL1, and activin A exhibit opposite activities in artritidies, namely, follistatin has a protective effect [74,75], while FSTL1 [24,76] and activin A [27,28,31,77] mainly worsen their development.

Interestingly, it has been established that activin A and its downstream signaling pathways play a role in regulating inflammation and immune response [80]. Therefore, the ratios between activin A, follistatin, and FSTL1 may reflect the inflammatory manifestation of KOA. The ambiguity of these associations represents one of the limitations of the present study. Confirmation of the observed relationships between follistatin/FSTL1 and activin A/FSTL1 ratios with KOA and elucidating their pathophysiological nature warrant further investigation.

Another novel observation in our study is the significantly higher plasma levels of activin A in women than men. The literature on this subject is virtually non-existent. However, information concerning the other observed inflammation-associated factors is available. For example, sex differences in several systemic inflammation markers have been observed in a large cross-sectional KORA study (641 men and 597 women aged 55–74) [81]. Similar to our method, this study used the BIA method for body composition assessment. The authors conclude that elevated cytokine levels in women vs. men are attributable to a more obese body composition in women, with “fat mass in % explained the highest percentage of the variability of circulating acute phase proteins”. Our data are in full accord with the above. In our sample, women, by all tested parameters, were significantly more obese than men (e.g., BMI, 31.51 ± 0.29 vs. 28.30 ± 0.24, *p* < 0.0001; FM/WT ratio: 0.39 ± 0.003 vs. 0.26 ± 0.003, *p* < 0.0001). Therefore, we believe that higher circulating levels of activin A in women vs. men in our population sample are also affected by a significantly higher rate of obesity in women.

Our study has several other limitations. The most notable one is its cross-sectional design which prevents drawing conclusions about the causality of the associations found. Longitudinal studies are required to establish the cause-and-effect relationships between KOA and the measured circulating factors as well as to evaluate their predictive nature. Another caveat is the lack of patient data on physical performance tests and clinical symptoms, including knee pain and joint function. As a result, we were unable to determine the possible associations of the measured parameters with functional impairment and severity of knee pain in KOA patients. The detection of these soluble factors in the synovial fluids of KOA patients is desirable but would not be possible in the control group and, therefore, not practical at this research project stage. Measuring pro-inflammatory factors and other soluble molecules in the blood for monitoring the onset, severity, and progression of KOA represents great interest because of the simplicity and availability of these assays. We should mention that although the effect of statistical adjustment for age was carried out on each of the study variables, some possible bias may occur due to the partial overlap of age in control and case groups, in which unaffected individuals tended to be younger than their affected counterparts. Finally, our findings were obtained in a single population; therefore, confirmation research in other populations is required to achieve generalizability of the results.

## 5. Conclusions

This study reports several novel findings with prognostic potential for KOA manifestation and progression. These include our findings that elevated SII, follistatin/FSTL1, and activin A/FSTL1 ratios were significantly and independently associated with KOA. We also report significant and independent associations between KOA and vaspin levels or FM/WT. These data corroborate multifactorial molecular mechanisms involved in KOA pathogenesis and suggest that several biomarkers can be used to predict the course of the disease accurately. Of interest, if these identified biomarkers play a role in KOA pathogenesis, they may serve as likely therapeutic targets for KOA patients.

## Figures and Tables

**Table 1:** Descriptive statistics of plasma marker levels and body composition measurements in individuals with KOA compared with control subjects.

Covariate	Control (*N* = 519)	KOA (*N* = 98)	*p*	*p* *
Age (years)	51.90 ± 0.38	56.16 ± 1.08	0.00002	
WHR	0.93 ± 0.003	0.94 ± 0.009	NS	NS
BMI (kg/m^2^)	29.62 ± 0.21	32.38 ± 0.51	0.000001	0.0003
FM/WT	0.32 ± 0.003	0.38 ± 0.008	3.63 × 10^−7^	0.0007
SMM/WT	0.31 ± 0.002	0.27 ± 0.005	0.000004	0.03
hs-CRP (mg/L)	1.50 ± 0.24	3.66 ± 0.84	0.001	0.001
SII (×10^9^/L)	455.01 ± 13.81	615.22 ± 40.96	0.000004	0.00002
Vaspin **	5.96 ± 0.04	6.44 ± 0.16	0.0003	0.001
Follistatin **	6.13 ± 0.03	6.39 ± 0.07	0.004	0.009
Activin A **	6.51 ± 0.05	6.84 ± 0.12	0.01	0.009
FSTL1 (ng/mL)	14.00 ± 0.23	13.03 ± 0.44	NS	NS
Follistatin (pg/mL)/FSTL1 (ng/mL)	47.42 ± 1.82	69.68 ± 7.71	0.00003	0.00006
Activin A (pg/mL)/FSTL1 (ng/mL)	96.10 ± 5.99	156.85 ± 21.96	0.0002	0.0007

The data are presented as mean ± standard error. N, sample size; KOA, knee osteoarthritis; WHR, waist–hip ratio; BMI, body mass index; FM/WT, fat mass/weight ratio; SMM/WT, skeletal muscle mass/weight ratio; hs-CRP, high-sensitivity C-reactive protein; and SII, systemic immune-inflammation index. *p* and *p* * are the results of the *t*-tests before and after adjustment for age and sex, respectively. ** Plasma levels of the vaspin, follistatin, and activin A are presented after log-transformation to approximate normality prior to analysis. The original measurements are given in Table S1. The mean values of these cytokines were compared by *t*-test (presented in the table) and by Mann–Whitney test (the corresponding *p*-values were 0.02, 0.003, and 0.02, respectively); and NS, not significant, *p* > 0.05.

**Table 2:** Multiple logistic regression analysis to explore the relationships between covariates and KOA (affected vs. unaffected).

	KOA Status, Affected (*N* = 98) vs. Controls (*N* = 519)		
Independent Covariate	OR (95% CI)	Β (SE)	*p*
FM/WT	1.56 (1.20–2.02)	0.44 (0.13)	0.0007
SII (×10^9^/L)	4.03 (2.23–7.27)	1.39 (0.30)	0.000003
Vaspin (pg/mL)	1.39 (1.15–1.68)	0.33 (0.09)	0.0005
Follistatin (pg/mL)/FSTL1 (ng/mL)	1.32 (1.02–1.70)	0.27 (0.13)	0.03
Activin A (pg/mL)/FSTL1 (ng/mL)	1.33 (1.01–1.75)	0.28 (0.13)	0.03

Data reported as odds ratio with 95% confidence intervals (OR (95% CI)), with corresponding beta coefficient and standard error (B (SE)); FM/WT, fat mass/weight ratio; and SII, systemic immune-inflammation index. At the initial stage of the study, the following independent variables were tested in stepwise-forward manners: FM/WT, SMM/WT, hs-CRP, SII, vaspin, follistatin/FSTL1, and activin A/FSTL1. All the variables in the analysis were adjusted for age and sex and standardized prior to statistical analysis. Only statistically significant terms are shown.

## Data Availability

Data is contained within the article and Supplementary Material.

## Supplementary Materials

The following supporting information can be downloaded at: https://www.mdpi.com/article/10.3390/biom13091315/s1, Table S1: Basic descriptive statistics of study variables by gender and disease status.
