# Supplementary material for: Pro-Inflammatory Biomarkers Combined with Body Composition Display a Strong Association with Knee Osteoarthritis in a Community-Based Study
Source: Biomolecules. 2023 Aug 28;13(9):1315. doi: 10.3390/biom13091315 (PMC10527309; doi:10.3390/biom13091315)
Supplement: Supplementary file 1 [file biomolecules-13-01315-s001.zip › biomolecules-2523399-supplementary.pdf]

Table S1. Basic descriptive statistics of study variables by gender and disease status.

	Males		Females	
**Variables**	**Control (N= 251)**	**KOA (N=30)**	**Control (N=268)**	**KOA (N=68)**
Age (y)	52.12±0.59	53.63±2.10	51.70±0.49	57.27±1.24
WHR	0.95±0.004	0.96±0.01	0.91±0.005	0.93±0.01
BMI (kg/m^2)	28.09±0.24	30.11±0.81	31.03±0.33	33.38±0.60
FM/WT	0.26±0.003	0.29±0.01	0.39±0.003	0.41±0.006
SMM/WT	0.36±0.002	0.34±0.007	0.25±0.002	0.24±0.003
hs-CRP (mg/L)	2.31±0.49	2.68±1.46	0.84±0.16	3.99±1.02
SII (×10^9/L)	459.79±21.55	604.42±90.71	450.32±17.47	618.51±46.31
Vaspin (pg/ml)	1705.10±351.54	3754.39±1474.85	1285.21±292.90	3255.21±870.10
Follistatin (pg/ml)	594.40±25.89	688.22±60.86	615.53±32.34	788.17±81.26
Activin A (pg/ml)	1018.46±76.90	1087.16±246.74	1343.31±109.92	2157.81±322.09
FSTL1 (ng/ml)	14.04±0.37	13.69±0.76	13.96±0.29	12.73±0.54

Data are presented as mean ± standard deviations; N, sample size; WHR, waist-hip-ratio; BMI, body mass index; FM/WT, fat mass/weight ratio; SMM/WT, skeletal muscle mass/weight ratio; hs-CRP, high-sensitivity C-reactive protein; SII, systemic immune-inflammation index.
